# Prototype foamy virus intasome aggregation is mediated by outer protein domains and prevented by protocatechuic acid

**DOI:** 10.1038/s41598-018-36725-1

**Published:** 2019-01-15

**Authors:** Nathan D. Jones, Randi M. Mackler, Miguel A. Lopez, Laura E. Baltierra-Jasso, Matthew P. Altman, Gayan Senavirathne, Kristine E. Yoder

**Affiliations:** The Ohio State University College of Medicine, Department of Cancer Biology and Genetics, 460 West 12th Ave, Columbus, OH 43210 USA

## Abstract

The integrase (IN) enzyme of retrovirus prototype foamy virus (PFV) consists of four domains: amino terminal extension (NED), amino terminus (NTD), catalytic core (CCD), and carboxyl terminus domains (CTD). A tetramer of PFV IN with two viral DNA ends forms the functional intasome. Two inner monomers are catalytically active while the CCDs of the two outer monomers appear to play only structural roles. The NED, NTD, and CTD of the outer monomers are disordered in intasome structures. Truncation mutants reveal that integration to a supercoiled plasmid increases without the outer monomer CTDs present. Deletion of the outer CTDs enhances the lifetime of the intasome compared to full length (FL) IN or deletion of the outer monomer NTDs. High ionic strength buffer or several additives, particularly protocatechuic acid (PCA), enhance the integration of FL intasomes by preventing aggregation. These data confirm previous studies suggesting the disordered outer domains of PFV intasomes are not required for intasome assembly or integration. Instead, the outer CTDs contribute to aggregation of PFV intasomes which may be inhibited by high ionic strength buffer or the small molecule PCA.

## Introduction

An essential step of the retroviral life cycle is integration of the viral genome to a host genome^1^. Following entry to a target cell, the genomic viral RNA is reverse transcribed to a cDNA. Integration is mediated by a poorly understood pre-integration complex (PIC) that minimally includes the viral protein integrase (IN) and the cDNA genome^2^. IN catalyzes the covalent joining of each end of the viral cDNA to the host genome, termed strand transfer. During an infection the PIC is an inherently transient protein complex. The complex must remain intact until it encounters host chromatin and completes integration. Following integration, the PIC disassembles to allow transcription of the viral genome^3^. Recombinant IN protein and DNA oligomers mimicking the cDNA termini may be assembled *in vitro* to an enzymatically functional complex termed an intasome^4^. There is little known about the assembly, disassembly, or stability of retroviral PICs or intasomes.

**Figure 1:**
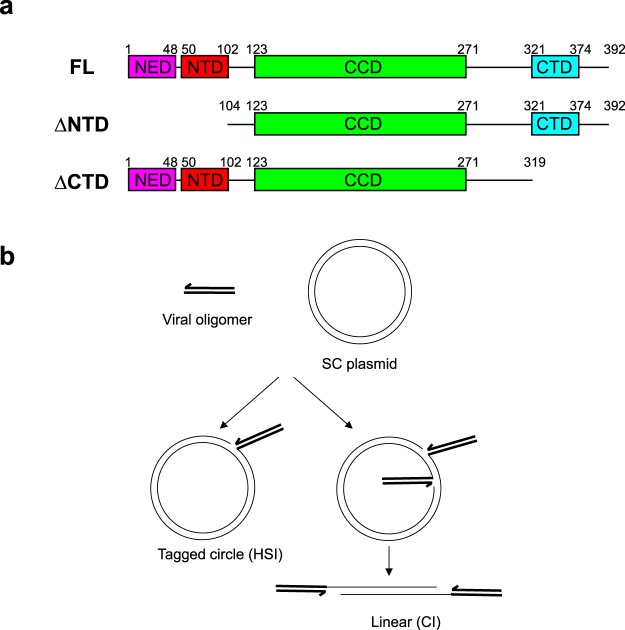
Cartoons of PFV IN and integration reaction products. (**a**) FL PFV IN is 392 amino acids. The truncation of NED (purple) and NTD (red) domains has the first 103 amino acids removed to generate ΔNTD. Truncation of the CTD (blue) deleted 73 amino acids. These truncation mutants preserved the PFV IN(K120E) and PFV IN(D273K) residues that direct monomers to the inner or outer positions of the intasome. (**b**) PFV intasomes catalyze the covalent joining of viral donor DNA oligomers to a supercoiled plasmid target DNA. Half site integration (HSI) is the joining of a single viral donor to the plasmid, generating a tagged circle. This integration reaction product has a nick at the point of joining that releases the supercoils. HSI products with fluorescently labeled viral donors include a single fluorophore. Concerted integration (CI) is more physiologically relevant and is the joining of two viral donors to the plasmid. This integration product relaxes to a linear product with the viral donor DNAs at the ends. There are two fluorophores present in CI products.

Prototype foamy virus (PFV) intasomes have been well characterized structurally^4,5^. These intasomes are a tetramer of PFV IN with two “inner” catalytically active subunits and two “outer” subunits that are not catalytically active, but are important for the architecture of the complex. Elegant structural studies revealed that point mutations PFV IN(K120E) and PFV IN(D273K) can direct monomers to the inner and outer subunits of the intasome, respectively^5^. PFV IN has four domains: an amino terminal extension domain (NED), amino terminal domain (NTD), catalytic core domain (CCD), and carboxyl terminal domain (CTD) (Fig. 1a)^6^. PFV IN(K120E) and PFV IN(D273K) are in linker regions that flank the CCD. Each domain of the inner monomers is bound to either target or viral DNA^4^. Only the CCDs of the outer monomers have been resolved in structures, leaving mysterious any possible roles for NED, NTD, or CTD of the outer monomers during integration.

Here we compare the integration activities of recombinant PFV intasomes with full length (FL) IN and mutant intasomes with truncations of the outer intasome monomers. We show that PFV FL intasomes display a limited lifetime under physiological conditions. Carboxyl terminal truncation mutants of the outer PFV IN monomers increase the activity and lifetime of intasomes. Deletion of the NED and NTD domains of the outer PFV IN monomers had minimal effects on intasome activity. We considered that the observed effects could be due to either disassembly or aggregation of intasomes. Increased salt concentration above physiological relevance or addition of small molecule protocatechuic acid (PCA) at physiologically relevant ionic strength increased the stability of FL intasomes. Precipitation experiments reveal that reduced stability is due to aggregation. Thus, high salt concentration or the addition of PCA is able to inhibit PFV intasome aggregation.

## Results

### Intasome outer CTDs reduce integration product accumulation

**Figure 2:**
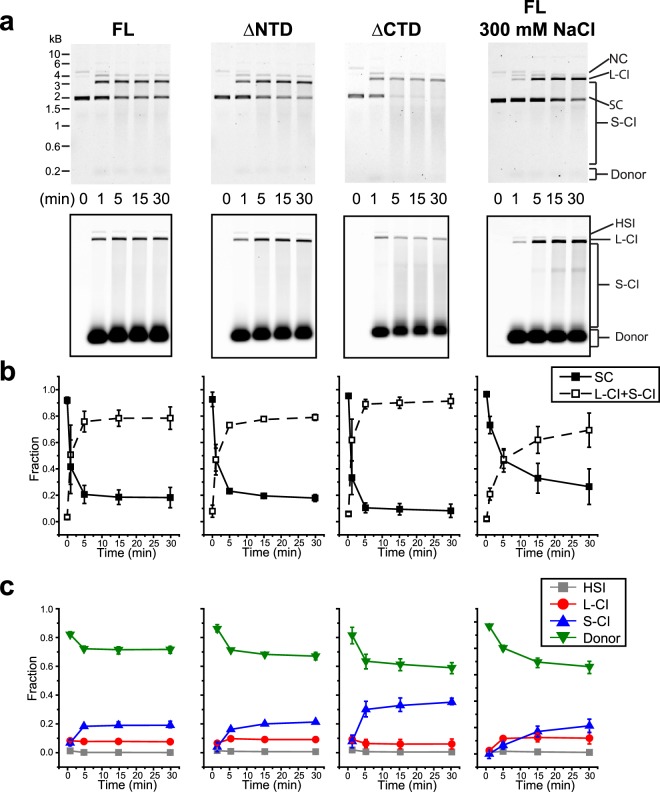
Time courses of integration by FL and truncation mutant PFV intasomes. PFV intasomes, including FL, ∆NTD, and ∆CTD, with Cy5 fluorescently labeled viral DNA were assayed for integration to a supercoiled plasmid over time. PFV FL intasomes were also assayed in the presence of 300 mM NaCl. (**a**) Integration products were separated by agarose gel electrophoresis and imaged for ethidium bromide (top panels) and Cy5 (bottom panels) fluorescence. (**b**) The ethidium bromide intensity of supercoiled plasmid is shown as the fraction of signal within each lane. The fraction of signal that is CI is the sum of the intensities of the linear band, the smear between linear and supercoiled bands, and the smear below the supercoiled band. (**c**) The relative Cy5 intensity within each lane is shown for the viral donor band, the linear CI product band, the HSI nicked circle band, and the smear of concerted integration products between the linear band and the viral donor. Experiments were performed at least three times with at least two independent intasome preparations. Error bars indicate standard deviation. NC, nicked circle. L-CI, linear CI products. SC, supercoiled plasmid. S-CI, smear CI products. Donor, viral donor DNA.

The PFV IN NED, NTD, and CTD of the outer intasome monomers were assayed for their roles during integration to a supercoiled plasmid. Recombinant PFV intasomes were assembled with Cy5 fluorescently labeled DNA oligomers mimicking the viral cDNA ends and purified by size exclusion chromatography (Supplementary Figure 1)^7^. When added to a supercoiled plasmid target DNA, PFV intasomes readily perform integration. The major product of PFV intasome integration is the joining of two viral DNA donors to the plasmid resulting in a linearized product, termed concerted integration (Fig. 1b, CI). A minor product is the integration of only one viral DNA donor yielding a tagged circle, called half site integration (HSI). The significance of HSI during infection is unclear. A third product may occur when the viral donor DNA is used as a target of integration, termed autointegration (AI). The products of integration assays *in vitro* may be resolved by agarose electrophoresis. Unreacted target DNA has the mobility of supercoiled plasmid, CI products appear as linear plasmid, and HSI products appear as nicked relaxed circles. More than one CI event to a single plasmid results in DNA fragments that are shorter than the linear plasmid and produce a smear of concerted integration products. Unreacted viral donor DNA and AI products have the fastest mobility. Ethidium bromide analysis reveals all DNA forms while fluorescent image analysis quantifies unreacted viral donor and integration products (Fig. 2a).

PFV intasomes assembled from FL inner and outer monomers were assayed for integration over time (Fig. 2). The ethidium bromide image indicates that the decrease in band intensity of the supercoiled plasmid is complete by 5 min of incubation (Fig. 2b, SC plasmid is 21% of the signal intensity within the lane). After 5 min, there is no further reduction of supercoiled plasmid. Similarly, the accumulation of ethidium bromide stained linear and smear CI products (L-CI and S-CI, respectively) reaches a maximum (76%) after 5 min. Cy5 fluorescence quantitation reveals that the major integration products for FL intasomes are CI visible as linear DNA and a smear (Fig. 2c). HSI products are minimal with PFV FL intasomes. Linear CI products are maximal at 1 min of incubation (8% of the fluorescent signal within the lane), while the smear CI products accumulate until 5 min (19%), indicating multiple integration events to a single plasmid. The fluorescent unreacted viral donor decreased until 5 min (72% of signal) inversely correlating with the accumulation of integration products, indicating that integration events lead to the smear CI products (Fig. 2c).

Integration kinetics of truncation mutants of the outer intasome subunits were compared to the FL intasomes (Fig. 2). Intasomes were assembled from full length PFV IN(K120E) as the inner monomers and truncation mutants of PFV IN(D273K) as the outer monomers. Deletion of the outer NED and NTD domains (for simplicity referred to as ∆NTD) resulted in CI kinetics similar to FL intasomes. The supercoiled plasmid decreased until 5 min (23%, Fig. 2b). Similarly, the accumulation of Cy5 fluorescent HSI (1%) and linear CI products (10%) were nearly equivalent to FL intasomes and complete at 5 min. However, the accumulation of smear CI products increased from 16% at 5 min to 21% at 10 min (p = 0.0041). Thus the PFV ∆NTD intasomes display nearly equal accumulation of integration products but are active longer compared to FL intasomes.

More dramatic differences were observed with deletion of the outer CTD domains (∆CTD) compared to FL intasomes. Ethidium bromide stained gel images suggest a complete disappearance of the supercoiled plasmid target by 5 min incubation (Fig. 2a). However, quantitation of the ethidium bromide stained supercoiled plasmid could be spurious due to smear CI products at the same mobility. The Cy5 fluorescent image revealed that significantly more PFV ∆CTD smear CI products (30%) accumulated at 5 min compared to FL (19%, p = 0.027). These data suggest that the presence of the outer intasome monomer CTDs reduces or inhibits integration. The data also suggest that the deletion of either the amino or carboxyl terminal domains on the outer monomers alters the yield of integration products.

### Catalytic activity of intasomes

The majority of PFV intasome products are CI with nearly undetectable HSI. However, all of the intasomes tested displayed fluorescent HSI products at 1 min of incubation (Fig. 2). This minor product disappeared by 5 min. It is possible that the HSI products were the substrate for a subsequent CI event. Alternatively, the data could suggest that HSI products captured at 1 min were an intermediate of a CI reaction. To test this second hypothesis, the time between strand transfer events was measured by magnetic tweezers. Previous measurement of the time between PFV FL intasome strand transfer events (τ_ST_) was 0.47 sec.^8^. Quantitation of the time between strand transfers for PFV FL intasomes for this study indicated 0.49 sec and was not significantly different from the previous report (Table 1). The time between strand transfers for PFV ∆NTD intasomes was 0.55 sec and PFV ∆CTD intasomes was 0.61 sec. Compared to FL intasomes these times are not significantly different (∆NTD p = 0.74, ∆CTD p = 0.49). This data argues against a model that the HSI events observed at 1 min are an intermediate of CI. It seems more likely that HSI events captured at 1 min are used as a target for CI events.

**Table 1:** Time between strand transfer events.

	τ_ST_
FL	0.49 ± 0.10 s (N = 38)
ΔNTD	0.55 ± 0.12 s (N = 15)
ΔCTD	0.61 ± 0.10 s (N = 47)
FL(PCA)	0.46 ± 0.14 s (N = 20)

The catalytic activity of the intasomes appeared to diminish after 5 min (Fig. 2). However, the intasomes showed differing amounts of CI products after 30 min (Fig. 2c). One explanation could be the catalytic rate differs between FL intasomes and truncation mutants. Early time points and a differential rate equation were used to determine and compare the initial catalytic activity (k_cat_) of the intasomes (Supplementary Figure 2). The initial k_cat_ was a function of the rate of supercoiled plasmid loss at one minute and the concentration of both intasome and plasmid (Supplementary Figure 2, Methods). The k_cat_ between intasomes were similar suggesting that deletion of the outer monomer domains did not affect the catalytic efficiencies of the complexes (Supplementary Figure 2b).

### PFV intasome affinity for target DNA

The increased integration product yield observed with PFV ∆CTD intasomes could be due to a greater affinity for supercoiled plasmid DNA. In this scenario the outer PFV IN CTDs may block target DNA binding or access to the active sites of the inner monomers. To test affinity for supercoiled plasmids, PFV intasomes were assembled with biotinylated viral donor DNA. The intasomes were added to supercoiled plasmid DNA and precipitated with streptavidin conjugated magnetic beads. DNA and proteins associated with the beads were evaluated by agarose electrophoresis and PAGE, respectively (Supplementary Figure 3). Control reactions indicated that intasomes effectively associated with the beads, but plasmid DNA alone did not (not shown). There was no significant difference between the amount of plasmid associated with FL, ∆NTD, or ∆CTD intasomes (∆NTD p = 1.0, ∆CTD p = 0.83). This data suggests that altered affinity for target DNA does not account for the observed differences in integration product accumulation.

### PFV intasome stability

**Figure 3:**
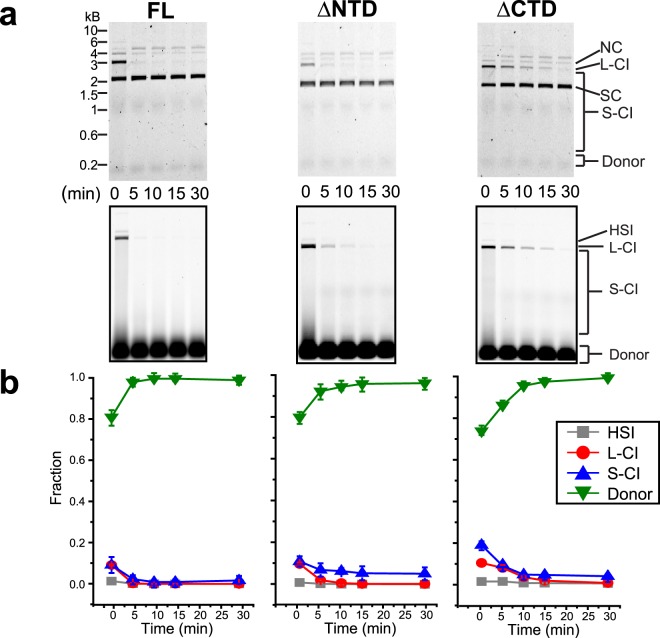
PFV intasome activity following preincubation. PFV intasomes with Cy5 labeled viral donor were incubated for variable time at 37 °C without target DNA. Following the preincubation, supercoiled plasmid target DNA was added to the reaction and incubated for a further 5 min. (**a**) Reaction products were separated by agarose gel electrophoresis stained with ethidium bromide (top panels) and imaged for Cy5 fluorescence (bottom panels). (**b**) Fluorescent products were analyzed and expressed as the fraction of fluorescent signal in each lane. Experiments were performed at least three times with at least two independent intasome preparations. Error bars indicate standard deviation. NC, nicked circle. L-CI, linear CI products. SC, supercoiled plasmid. S-CI, smear CI products. Donor, viral donor.

The PFV FL intasomes appeared to have little or no activity following incubation for 5 min (Fig. 2). In contrast, both truncation mutant intasomes displayed some increase of CI products after 5 min. The loss of FL intasome activity could be due to a loss of stability, either through disassembly or aggregation. To test for stability effects on integration activity, fluorescently labeled intasomes were incubated at 37 °C for variable time without target DNA. After this preincubation, target DNA was added and the accumulation of integration products in 5 min was assayed (Fig. 3a). Without preincubation, FL intasomes display detectable CI events as linear and smear products. However, these products are not apparent after 5 min preincubation. Similarly, the viral donor DNA is reduced to 80% of the total fluorescent signal without a preincubation, but is nearly 100% at all preincubation times (Fig. 3b).

In contrast, PFV ∆NTD intasomes were more resistant to preincubation. The accumulation of PFV ∆NTD intasome integration products was approximately equal to FL intasomes without preincubation. However, PFV ∆NTD intasomes retained 2% linear CI products after a 5 min preincubation and maintained the ability to generate a small amount of smear CI products (5%) even after a 30 min preincubation (Fig. 3b). The concomitant 95% lane intensity of viral donor suggests that the smear is due to CI events.

The PFV ∆CTD intasomes appeared to retain more linear CI over time compared to FL or ∆NTD intasomes (Fig. 3). The ∆CTD intasome linear CI products continued to accumulate after 10 min preincubation. The smear CI products reached a plateau (3%) after 10 min preincubation, but remained constant through 30 min preincubation, similar to the ∆NTD intasomes (Fig. 3b). Together this data suggests that FL intasomes are the most sensitive to preincubation at 37 °C, while the truncation mutant intasomes retain integration activity even after 30 min preincubation.

**Figure 4:**
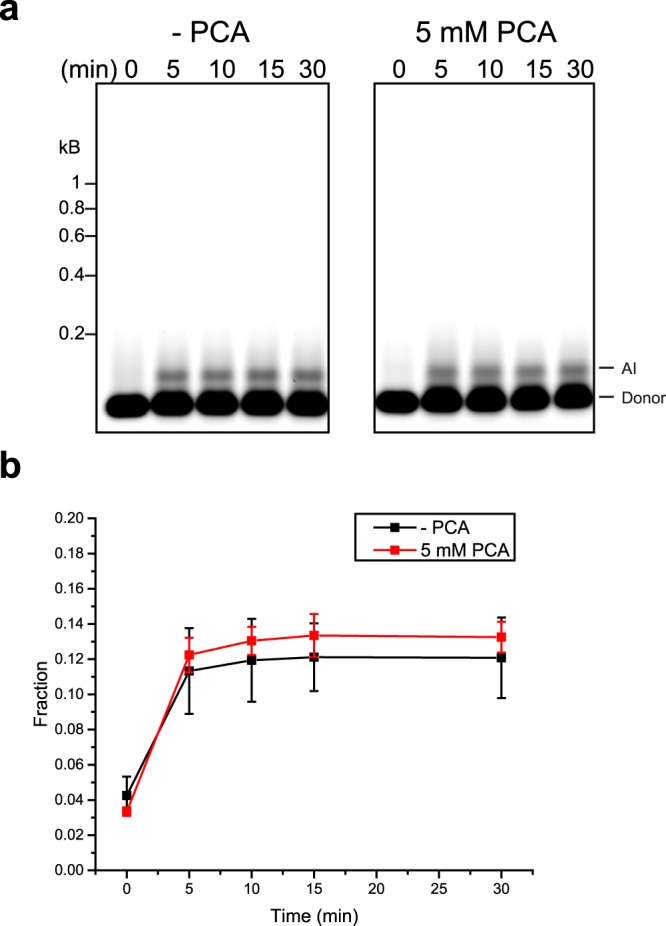
PFV intasome autointegration activity. PFV intasomes with Cy5 labeled viral donor DNA were incubated for variable time at 37 °C in the absence or presence of 5 mM PCA. (**a**) Reaction products were separated by agarose gel electrophoresis and imaged for Cy5 fluorescence. (**b**) Fluorescent products were analyzed as a fraction of total fluorescent signal in each lane. There appears to be similar AI product accumulation regardless of PCA addition.

HSI and CI products appeared to be absent when FL intasomes were subjected to a preincubation. However, the intasomes could still retain undetected AI activity. The AI activity of FL intasomes was evaluated by incubation at 37 °C without added target DNA (Fig. 4). Similar to results seen with added supercoiled plasmid target DNA, the AI activity of FL intasomes was static after 5 min (Figs 2 and 4).

**Figure 5:**
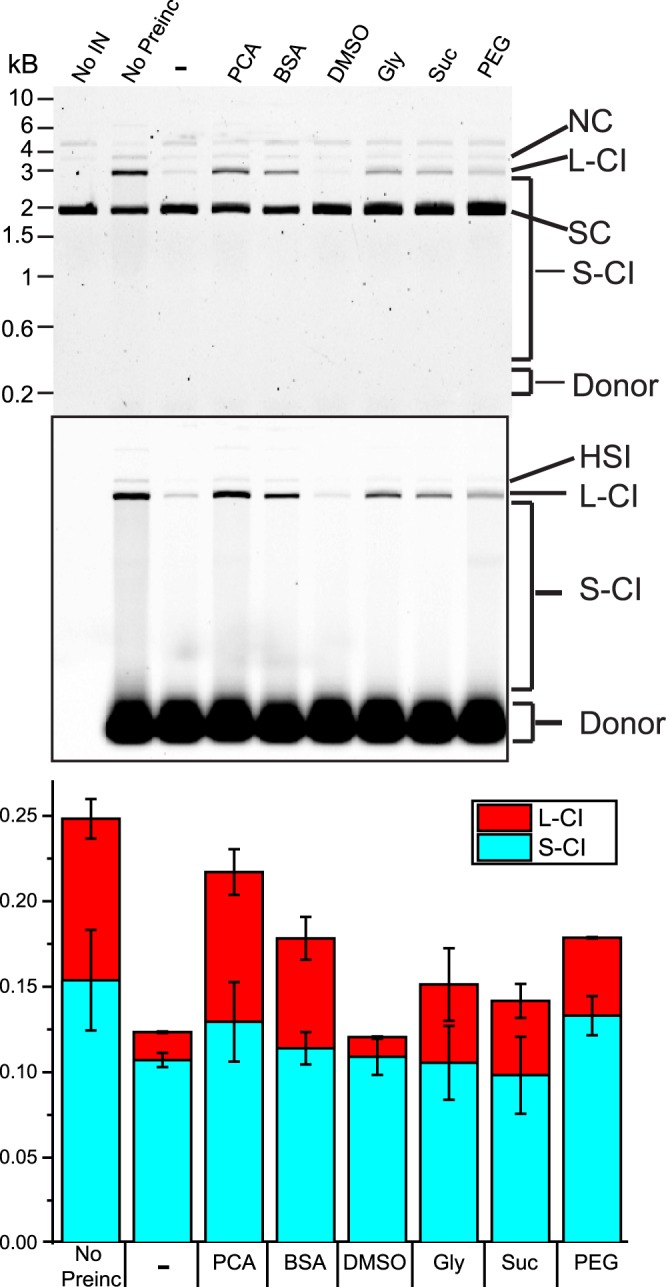
Rescue of PFV intasome activity by additives present during preincubation. PFV FL intasomes were incubated at 37 °C for 5 min. Following the preincubation, target DNA was added and reactions were incubated for an additional 5 min at 37 °C. Additives included in the reactions were: 5 mM PCA (PCA), 0.1 mg/ml acetylated BSA (BSA), 5% DMSO (DMSO), 10% glycerol (Gly), 10% sucrose (Suc), and 10% PEG 6000 (PEG). Control reactions include no preincubation (No preinc) or no addition of an additive (−). Fluorescent products were analyzed and expressed as the fraction of fluorescent signal in each lane (bottom graph). Experiments were performed at least three times with at least two independent intasome preparations. Error bars indicate standard deviation.

Historically, additives have been included in retroviral integration reactions to enhance activity. These additives include BSA, DMSO, glycerol, or PEG 6000^9–13^. Their ability to enhance the stability of PFV FL intasomes was assayed (Fig. 5). PFV FL intasomes were preincubated for 5 min in the presence of the additives. PCA, commonly used in oxygen scavenging systems, and sucrose were included in addition to the previously reported additives. Following the preincubation, target DNA was added and the reactions were incubated for an additional 5 min.

Preincubation of PFV FL intasomes for 5 min significantly reduced the yield of CI products (Fig. 5). Smear CI products were reduced from 15% of the fluorescent signal to 10.5%, the linear CI products were reduced >5 fold from 9.4% to 1.6% (Fig. 5, bottom panel). Addition of 5% DMSO had no effect on the accumulation of integration products. The other additives displayed a greater effect on the accumulation of linear CI products compared to smear CI products. The presence of 10% glycerol, 10% sucrose, or 10% PEG 6000 increased the observed linear CI products to a similar extent (4.6%, 4.3%, and 4.6%, respectively). Inclusion of 0.1 mg/ml acetylated BSA was better able to enhance linear CI following a preincubation (6.4%). However, the additive that showed the greatest ability to rescue linear CI products following preincubation was 5 mM PCA (8.8%). Unlike the other additives tested, PCA and acetylated BSA are negatively charged. PCA showed no effect on the time between strand transfer reactions of FL intasomes when tested with magnetic tweezers (Table 1). This small molecule has not previously been reported as an effective crowding agent or to have a stabilizing effect on protein complexes.

**Figure 6:**
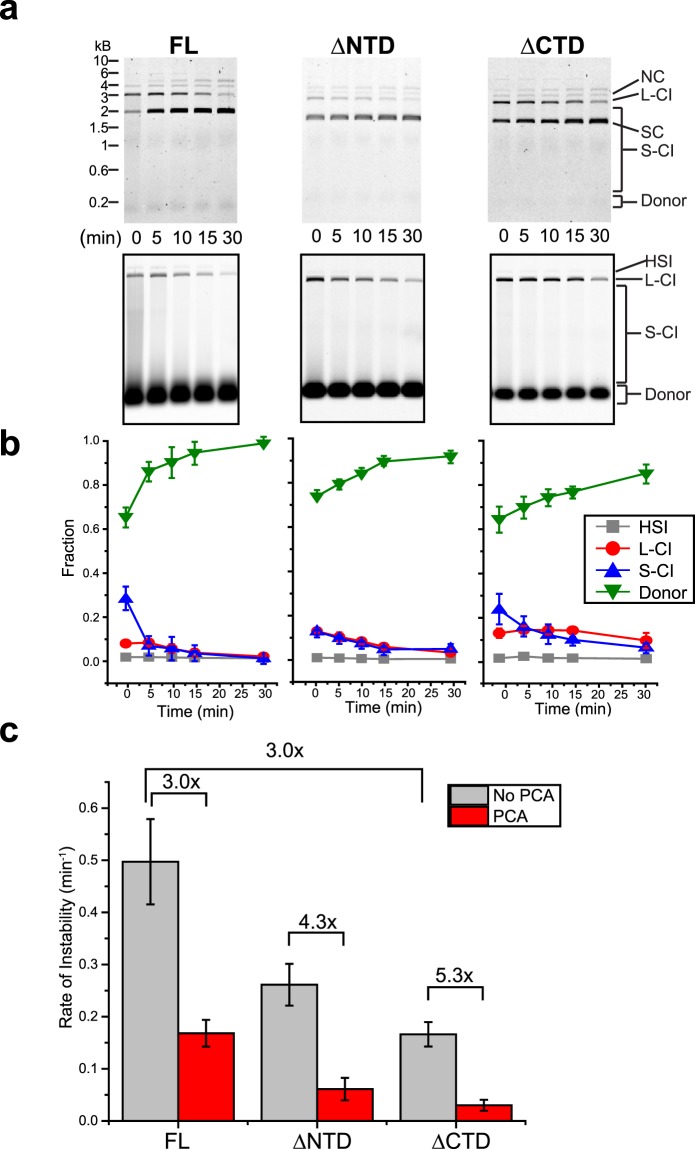
PFV intasome activity following preincubation in the presence of PCA. PFV intasomes with Cy5 labeled viral donor DNA were incubated for variable time at 37 °C with PCA but without target DNA. Following the preincubation, supercoiled plasmid target DNA was added to the reaction and incubated for a further 5 min. (**a**) Reaction products were separated by agarose gel electrophoresis stained with ethidium bromide (top panels) and imaged for Cy5 fluorescence (bottom panels). (**b**) Cy5 fluorescent products were analyzed and expressed as the fraction of fluorescent signal in each lane. (**c**) Quantitation of the rate of increase in Cy5 donor with and without PCA (rate of instability). The addition of PCA correlates with a decrease in the rate of donor accumulation. Reactions were performed in the presence of 5 mM PCA. Experiments were performed at least three times with at least two independent intasome preparations. Error bars indicate standard deviation. NC, nicked circle. L-CI, linear CI products. SC, supercoiled plasmid. S-CI, smear CI products. Donor, viral donor.

The effects of PCA were assayed with FL and truncation mutant intasomes (Fig. 6). Intasomes were incubated at 37 °C for variable time in the presence of PCA without target DNA. Following the preincubation, target DNA was added and reactions were incubated for an additional 5 min (Fig. 6a). All intasomes show greater CI activity in the presence of PCA (Fig. 6b). HSI products were negligible (<1%) for all intasomes. The viral donor DNA intensity curves show a marked difference between with and without PCA (compare Fig. 4b to Fig. 6b). PFV FL intasomes were inactive after 5 min preincubation in the absence of PCA, but retained CI activity after 15 min preincubation in the presence of PCA. The FL intasome smear CI products were the most affected without preincubation, accumulating 10% and 28% in the absence or presence of PCA, respectively. Although PCA appeared to enhance the CI activity of FL intasomes at longer incubation times, it had no effect on the accumulation of AI products over time (Fig. 4).

PCA was also able to enhance the stability of truncation mutant intasomes. PFV ∆NTD intasomes were minimally active displaying only smear CI products (5%) after 10–30 min preincubation (Fig. 6b). In the presence of PCA, linear CI products (3%) continued to accumulate after 30 min preincubation and smear CI products steadily decreased from 13% to 5% between 0 and 15 min preincubation. The smear CI products remained constant between 15 and 30 min preincubation. PFV ∆CTD intasome linear CI products were also affected by the addition of PCA during preincubation. The linear CI products were 9% of the lane intensity after 30 min preincubation, but were not present at this time point without PCA. Smear CI products were increased between 0–30 min preincubation when PCA was added.

In order to evaluate the effect of PCA on intasome stability, the fluorescent viral donor DNA data were fit to an exponential curve to obtain k, the rate of unreacted viral donor accumulation over time (Fig. 6c, referred to as rate of instability). Unreacted viral donor DNA is a measure of intasomes that do not participate in integration. This inactivity may be due to instability, either disassembly or aggregation. This analysis revealed that FL intasomes are 3 fold less active than ∆CTD intasomes in the absence of PCA. The addition of PCA increased the FL intasome activity 3 fold. Interestingly, PCA was able to stabilize or increase the activity of ∆CTD intasomes (5.3 fold) to a greater extent than the increase of FL intasome activity. This data suggests that the mechanism of ∆CTD intasome stability and activity is at least partially distinct from PCA mediated effects.

### Non-physiological high salt concentration

Previous studies of PFV intasomes have included buffers with salt concentrations higher than physiologically relevant^5^. PFV intasomes appear to be relatively stable in conditions of higher ionic strength and are purified in the presence of 320 mM NaCl^7^. The activity of FL intasomes over time was measured in the presence of 300 mM NaCl (Fig. 2). The increased ionic strength of the buffer led to altered integration kinetics. The band of supercoiled plasmid was slower to decrease in intensity and did not decrease to the same extent (26%) seen under more physiological conditions (18%) (Fig. 2b). The fluorescent linear CI products were slightly increased and reached saturation at 5 min in 300 mM NaCl (Fig. 2c). However, the fluorescent smear CI products continued to accumulate throughout the 30 min incubation. The FL intasome rate of integration was dramatically altered with higher salt conditions (Fig. 3). After 1 min incubation, the rate of integration was 8.75 fold faster in the presence of 110 mM NaCl compared to 300 mM NaCl. However, at subsequent times the FL intasomes displayed a slower rate of integration in physiological ionic conditions. These data suggested that high salt concentration alters FL intasome activity kinetics, but allows a longer intasome lifetime.

**Figure 7:**
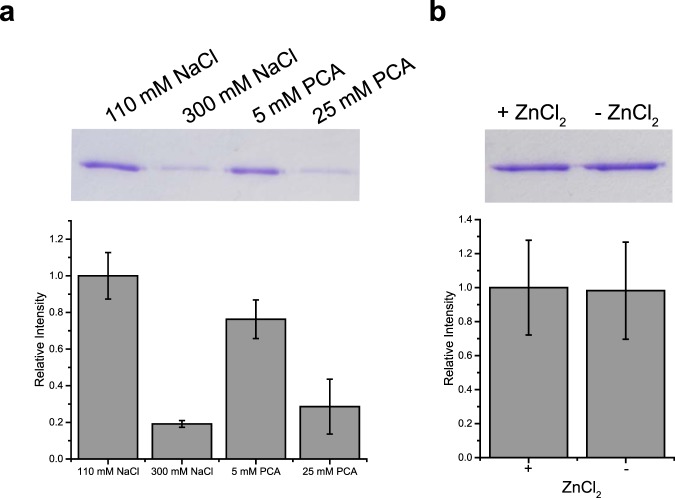
PFV intasome aggregation. PFV FL intasomes were incubated at 37 °C for 5 min. The reactions were then centrifuged to pellet aggregates. Pellets were analyzed by SDS-PAGE stained with Coomassie blue. (**a**) Reactions included 110 mM NaCl with no PCA, 300 mM NaCl with no PCA, 110 mM NaCl with 5 mM PCA, or 110 mM NaCl with 25 mM PCA. Precipitated PFV IN is expressed relative to reactions with 110 mM NaCl and no PCA. (**b**) FL intasomes were incubated in the presence or absence of ZnCl_2_. Precipitated PFV IN is expressed relative to reactions with ZnCl_2_ present. Experiments were performed at least three times with at least two independent intasome preparations. Error bars indicate standard deviation.

The instability of PFV FL intasomes could be due to disassembly or aggregation. To test the propensity to aggregate, FL intasomes were incubated for 5 min at 37 °C and immediately centrifuged at 4 °C for 30 min. Pellets were analyzed by SDS-PAGE for precipitated IN (Fig. 7). Comparison of FL intasome aggregation at variable salt concentration reveals 5.6 fold less IN precipitation at 300 mM NaCl. The addition of 5 mM PCA was able to prevent some aggregation, reducing the precipitate to 76%. The effect of PCA was concentration dependent with 25 mM PCA reducing the precipitated protein to 24%. The ability of zinc ions to participate in intasome aggregation was also evaluated. FL intasomes in the presence of 110 mM NaCl with or without ZnCl_2_ were found to display no difference in aggregation. These data suggest that incubation at 37 °C promotes aggregation of PFV FL intasomes. This aggregation may be prevented by conditions of increased ionic strength or the addition of small molecule PCA.

## Discussion

The outer domains of the PFV intasome were not visualized in a crystal or cryo-EM structure^4,5^. This observation could suggest that these domains play no role in catalysis. A previous study of PFV intasomes compared FL IN to CCD only outer monomers^14^. This study was performed without the use of PFV IN(K120E) and IN(D273K) point mutations^5^. Instead the authors employed an elegant purification scheme^14^. The study concluded that the outer amino and carboxyl terminal domains are not necessary for intasome assembly or integration. Using point mutations to target truncation mutants to the outer intasome monomers, we were able to confirm and extend the previous report.

PFV IN NED and NTD domains from the outer monomers displayed the least effects on intasome activity compared to FL intasomes. Deletions of the outer PFV IN CTDs had more dramatic effects on the accumulation of integration products. The CTDs of retroviral INs have been implicated in tethering intasomes to targets, although it has not been possible in most cases to assign this interaction to inner or outer monomers. The murine leukemia virus IN CTD interacts with the host BET proteins and avian leukosis virus IN CTD similarly interacts with the FACT complex to tether intasomes to chromatin^15–17^. PFV IN CTDs of both inner and outer monomers appear able to bind the amino terminus of the host H2A histone protein^5^. In addition to protein binding, retroviral IN CTDs have DNA binding activity^18^. We found that the outer monomer CTDs or NTDs have no effect on intasomes binding to supercoiled plasmid DNA.

Deletion of the outer domains seems to enhance the stability of PFV intasomes at 37 °C. The relatively low stability of FL intasomes appears due to aggregation. It has previously been shown that protein interface regions contribute to aggregation more than other protein surfaces^19^. The IN amino and carboxyl terminal domains play multiple roles in binding viral and cellular DNA, other IN monomers, and host proteins^4,5,15–18,20^. The surfaces of outer monomers may play a significant role in aggregation of FL intasomes. This type of aggregation would not be predicted to occur *in vivo* since cellular infection includes a single intasome as opposed to the nM concentrations in biochemical reactions.

Since the outer monomer amino and carboxyl terminal domains appear dispensable for catalysis, more stable retroviral intasomes assembled with truncation mutants may advance studies of these complexes. To date, point mutations that direct IN to the inner or outer subunits have only been described for PFV^5^. The purification method previously described for PFV intasomes with only CCD outer monomers may be an alternative for other retroviral intasomes^14^. Alternatively, small molecule PCA may prevent aggregation of FL retroviral intasomes at physiologically relevant ionic strength conditions.

Although increased concentration of NaCl was also able to prevent aggregation of PFV FL intasomes, study of intasome activity with supercoiled plasmid DNA is not likely to produce physiologically relevant data. The kinetics of CI product accumulation with FL intasomes at 110 mM or 300 mM NaCl were exceptionally different (Fig. 2). Increased NaCl concentration increases DNA twist by 0.03°/bp, compacting the supercoiled DNA, and possibly generating a less favored target for PFV IN^21,22^. Physiologically relevant salt concentration allows loose dynamic configurations of circular DNA^21^. Although retroviral INs may retain catalytic activity at higher salt concentrations, the effects of ions on target DNA conformation should be considered. Increased ionic concentration allows for closer interactions between segments of a negatively charged target DNA molecule, possibly limiting interfaces for PFV integration. In contrast, truncation mutants or PCA do not alter the target DNA conformation at physiologically relevant conditions suggesting that these approaches will yield more relevant data than studies performed under non-physiologic conditions.

## Methods

### Subcloning PFV IN truncation mutants

The point mutations for inner PFV IN(K120E) and outer PFV IN(D273K) were expressed as previously described for wild type PFV IN^23,24^. Truncation mutants were engineered to the intasome outer monomers with the point mutation PFV IN(D273K). The amino terminal 103 amino acids were deleted to generate PFV IN(∆NED,∆NTD, D273K). This construct includes the unstructured linker region between the PFV IN NTD and CCD, including the residue K120. Primers KEY900 5′ GGGACCCGGGGCTTCCAACAAAGCCTCTGGTCCTATTC 3′ and KEY620 5′ TTCCAAATGATCCATTGTTGCAG 3′ amplified the PFV IN truncation. The PCR product was subcloned into XmaI and AflII sites. The carboxyl terminal 73 amino acids were deleted to generate PFV IN(∆CTD, D273K). This truncation mutant includes the linker region with an alpha helix between CCD and CTD, importantly the PFV IN(D273K) mutation is preserved. Primers KEY674 5′ GGATCGAGATCTCGATCCCGCG 3′ and KEY899 5′ GCCGGATCCTCAAACAACAGGAGACCAGGAACGAGAGG 3′ amplified this fragment of PFV IN. The PCR product was subcloned into XbaI and BamHI sites.

### Purification of PFV integrase and intasomes

PFV IN was purified as previously described^23,24^. Proteins purified include full length (FL) PFV IN, PFV IN(K120E), PFV IN(∆NED, ∆NTD, D273K), and PFV IN(∆CTD, D273K). Point mutations and truncation mutants of PFV IN were purified with the same method as full length PFV IN.

Intasomes were assembled as described^7^. Briefly, 120 µM PFV IN, 50 mM Bis-tris propane, pH 7.5, 500 mM NaCl, and 50 µM vDNA were combined in 120 µL final volume. The assembly was placed in 6–8 kDa molecular weight cut off (MWCO) dialysis tubing in 1 L 20 mM Bis-tris propane, pH 7.5, 200 mM NaCl, 2 mM DTT, 25 µM ZnCl_2_. Intasomes were assembled at 18–22 °C overnight. The intasome precipitate was solubilized with the addition of NaCl to a final concentration of 320 mM. Solubilized intasomes were fractionated by Superose 12 10/300 GL size exclusion chromatography (SEC, GE Healthcare) in running buffer 20 mM Bis-tris propane, pH 7.5, 320 mM NaCl, 10% glycerol. Fractions were analyzed by SDS-PAGE, snap frozen in liquid nitrogen, and stored at −80 °C. Frozen intasomes begin to lose integration activity after one year in storage. For simplicity the intasomes are referred to only by the outer monomer identity, such as PFV FL, PFV (∆NTD) or PFV (∆CTD). Fluorescently labeled viral donor DNA was generated by annealing DNA oligomers KEY616 5′ ATTGTCATGGAAT*TTTGTATATTGAGTGGCGCCCGAACAG 3′ and KEY675 5′ CTGTTCGGGCGCCACTCAATATACAAAATTCCATGACA 3′. These DNA oligomers yield a viral donor DNA mimicking a 3′ preprocessed viral DNA end. The Cy5 fluorophore was added to KEY616 at T13, an internal amino-T. To generate biotinylated intasomes, a biotin was added to the 5′ end of KEY675.

### Integration reactions

PFV intasome integration reactions were performed as described with minor modifications^7^. Reactions included 10 mM bis-tris propane, pH 7.5, 110 mM NaCl, 5 mM MgSO_4_, 4 µM ZnCl_2_, 10 mM DTT, 26 nM intasome, and 1.8 nM supercoiled plasmid pMP2. Time course reactions were in a total volume of 67.5 µL with 15 µL removed at each time point. Preincubation experiments were incubated at 37 °C for 5 min before the addition of target DNA. Small molecules added to stabilize intasomes were 5 mM or 25 mM protocatechuic acid (PCA, MP Biochemicals or 3,4 dihydroxybenzoic acid, Sigma Aldrich), 0.1 mg/ml acetylated BSA (Promega), 5% DMSO (Sigma Aldrich), 10% glycerol (Sigma Aldrich), 10% sucrose (>99.9%, Fisher Scientific), 10% polyethylene glycol 6000 (Sigma Aldrich) in 15 µL reaction volume. Reactions with PCA included 30 mM bis-tris propane, pH 7.5 (Sigma Aldrich). Reactions were performed with two independent preparations of all intasomes. Experiments were performed at least three times, unless noted. Agarose gels were scanned for Cy5 and ethidium bromide fluorescence by a Typhoon 9410 variable mode fluorescent imager (GE Healthcare). Lane intensities were analyzed using GelAnalyzer software (Lazar Software). Smear signal was quantified as cumulative signal above the gel background. The differential rate equation for the intial change in concentration of supercoiled plasmid [SC] can be written as:

1 $${(\frac{d[SC]}{dt})}_{initial}=-\,{k}_{cat}{[SC]}_{0}{[IN]}_{0}$$

In the experiment [IN]_0_ and [SC]_0_ are the initial concentrations, therefore constant. The k_cat_ is calculated with Eq. 1 and the decay curves of SC from ethidium bromide stained gels. The left term can be calculated by the first derivative of the initial decay of [SC]. The [SC] decay curves were fit with exponential decay trend lines. K_cat_ is in units of (1/(nM*min)). The rate of instability was determined by fitting an exponential decay function to Cy5 viral donor DNA intensity over time: *Y* = *A*(1 − *e*^*−kt*^), where k is the value of the rate of instability.

### Single molecule magnetic tweezers

Experiments were performed as previously described^8^. Briefly, flow cells were engineered with glass cover slides attached to an aluminum chip. Prior to attachment the glass slides were treated with (3-Aminopropyl) triethoxysilane followed by a 1:100 mixture of Biotin-PEG SVA to mPEG-SVA (Invitrogen). Plasmid pET-29a was digested with EcoRI and SphI yielding a 4967 bp linear fragment, where biotin and digoxigenin-labeled ends were added. Neutravidin (500 µM, Invitrogen) was injected in the flow cell at a rate of 8 μl/min, followed by the digested product. Tosylactivated M-280 SPM Dynabeads (ThermoFisher Scientific) were coated with anti-digoxigenin antibodies and injected into the flow cell. The bound DNA was washed extensively with integration buffer. Introduction of supercoils used two 1 cm^3^ rare earth magnets (Neodynium, Magcraft). For PFV integration experiments, the DNA was wound clockwise with 10 complete turns of the magnets to induce negative supercoils. The SPM beads were imaged using a 530 nm LED lamp (Thorlabs), a 40X Olympus oil immersion objective and images collected on a 1024 × 1024 pixel charge coupled device (CCD) camera (Grasshopper Express 1.0 MP Mono FireWire 1394b) at a frame rate of 100 msec for at least 1800 sec. The time measured between the two strand transfer events is defined as τ_ST_.

### Binding experiments

PFV intasomes with biotinylated viral donor DNA were added to supercoiled plasmid pMP2 in 50 mM HEPES, pH 7.5, 110 mM NaCl, 1 mM DTT, 10% glycerol, 0.1% Tween-20, 1 µg/ml acetylated BSA, and 0.03 mM EDTA in a total volume of 35 µL. Reactions were incubated first on ice for 20 min and then at ambient temperature for 30 min. Streptavidin conjugated magnetic beads (Dynabeads M-280 streptavidin, Thermo Fisher) were added and reactions were incubated at ambient temperature for 1 hour with rotating. Beads were washed three times with reaction buffer. Following the final wash, beads were resuspended in PBS and boiled for 10 min. Half of the reaction was analyzed by 12% denaturing PAGE and half was analyzed by 1% agarose gel stained with ethidium bromide. Reactions were performed twice with two independent preparations of intasomes. Agarose gels were scanned for ethidium bromide fluorescence by a Typhoon 9410 variable mode fluorescent imager (GE Healthcare). Coomassie blue stained SDS-PAGE gels were imaged with an Epson Perfection V37 scanner. Lane intensities were analyzed with GelAnalyzer software (Lazar Software).

### Aggregation experiments

25 nM PFV FL intasomes in 10 mM bis-tris propane, pH 7.5, 5 mM MgSO_4_, 4 µM ZnCl_2_, and 10 mM DTT in a final volume of 100 µL were incubated at 37 °C for 5 min. Samples included the indicated amount of NaCl or PCA. Samples with PCA were in the presence of 110 mM NaCl. Following incubation, the samples were centrifuged at 18,000 *g* for 30 min at 4 °C. Pellets were resuspended in 1X Laemmli buffer, boiled, and analyzed by 12% SDS-PAGE. Gels were stained with Coomassie brilliant blue and scanned with a Sapphire biomolecular imager (Azure). The experiment was repeated three times with at least two independent intasome preparations. Lane intensities were analyzed with GelAnalyzer software (Lazar Software).

## Electronic supplementary material

Supplementary Information

## Data Availability

All data generated or analyzed during this study are included in this published article (and its Supplementary Information files).
