# Supplementary material for: Prototype foamy virus intasome aggregation is mediated by outer protein domains and prevented by protocatechuic acid
Source: Sci Rep. 2019 Jan 15;9:132. doi: 10.1038/s41598-018-36725-1 (PMC6333793; doi:10.1038/s41598-018-36725-1)
Supplement: Supplementary file 1 — Supplementary Information [file 41598_2018_36725_MOESM1_ESM.pdf]

Prototype foamy virus intasome aggregation is mediated by outer protein domains and prevented by protocatechuic acid

Nathan D. Jones[1,†], Randi M. Mackler[1,†], Miguel A. Lopez Jr.[1,†], Laura E. Baltierra-Jasso[1], Matthew P. Altman[1], Gayan Senavirathne[1], and Kristine E. Yoder[1,*]

[1]The Ohio State University College of Medicine, Department of Cancer Biology and Genetics, 460 West 12th Ave. Columbus, OH, 43210

[†]authors contributed equally

*Correspondence to yoder.176@osu.edu

Supplementary Figure 1

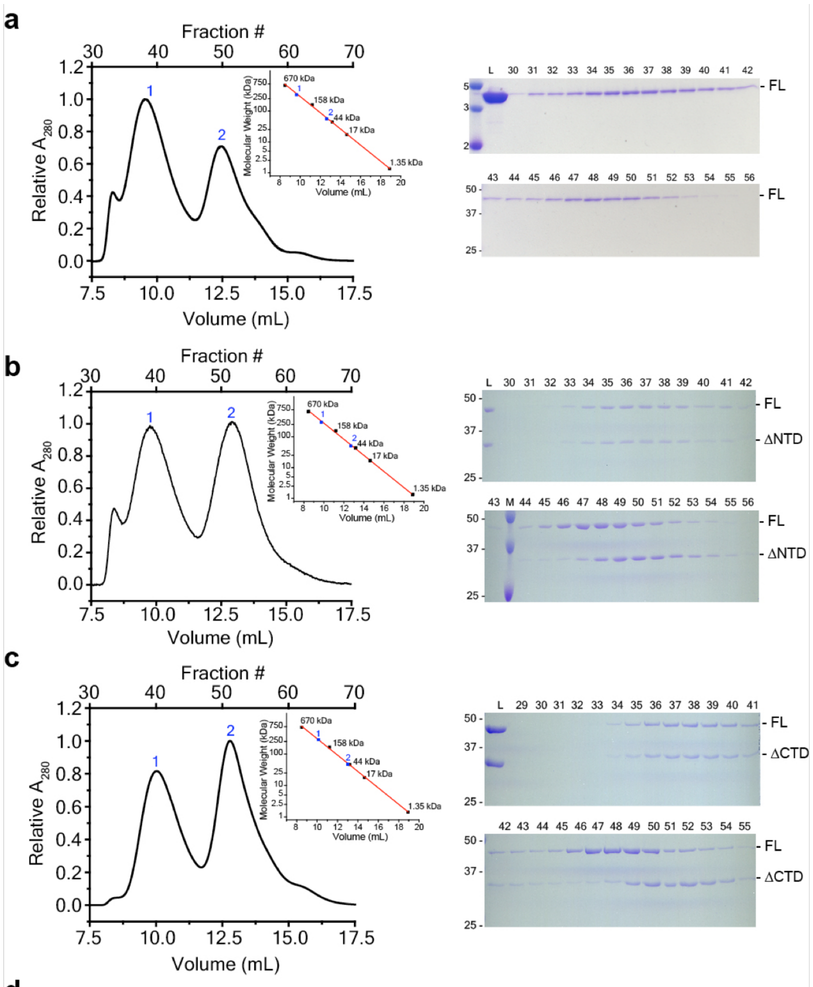

d

IN	MW (kDa)
FL	44.4
ΔNTD	32.5
ΔCTD	36.2
vDNA	23.6

Intasome	Expected MW (kDa)	Experimental MW (kDa) - Peak 1	Experimental MW (kDa) - Peak 2
FL	224.9	334.1	55.7
ΔNTD	201.1	299.6	45.1
ΔCTD	208.5	272.2	44.8

Supplementary Figure 1. Purification of FL and Truncated Hybrid PFV intasomes. (a) FL, (b) ΔNTD and (c) ΔCTD intasomes were purified by size exclusion chromatography (SEC) using a Superose 12 gel filtration column. Two distinct peaks are observed that correspond with intasomes (1), and unassembled protein monomer (2). SDS-PAGE gels for (a) WT, (b) ΔNTD and (c) ΔCTD SEC fractions are shown. L is load. Molecular weight (MW) standards shown on left in kDa. Insets show elution peaks relative to MW SEC standards. Truncated hybrid intasomes (b,c) were confirmed by coelution of FL and truncated proteins in peak (1) compared to asynchronous elution in peak (2). (d) Table of expected and experimental MWs in kDa. Observed molecular weight differences are likely a result of the asymmetrical nature of the PFV intasome.

Supplementary Figure 2

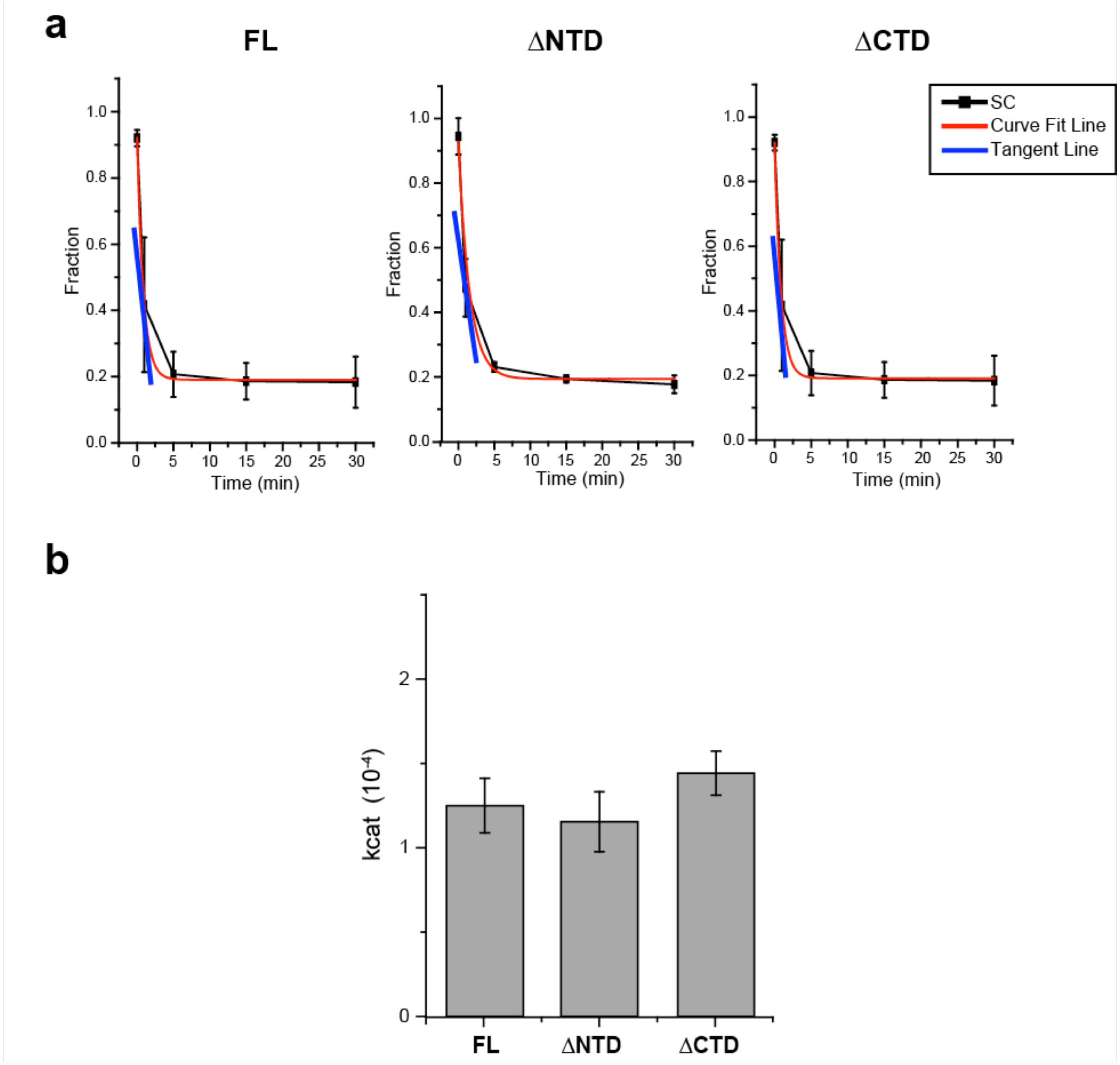

Supplementary Figure 2. Initial kinetics of PFV integration. (a) Data fitting for the loss of supercoiled DNA with FL, ΔNTD and ΔCTD intasomes. Data was fit with exponential decay curves (curve fit line). The first derivative was found and evaluated at the one minute time point (tangent line). (b) Using Eq. 1, the initial k_{cat} was solved for each time course. The k_{cat} reveals no significant difference between the initial rates of supercoiled DNA decrease between the FL, ΔNTD and ΔCTD intasomes.

Supplementary Figure 3

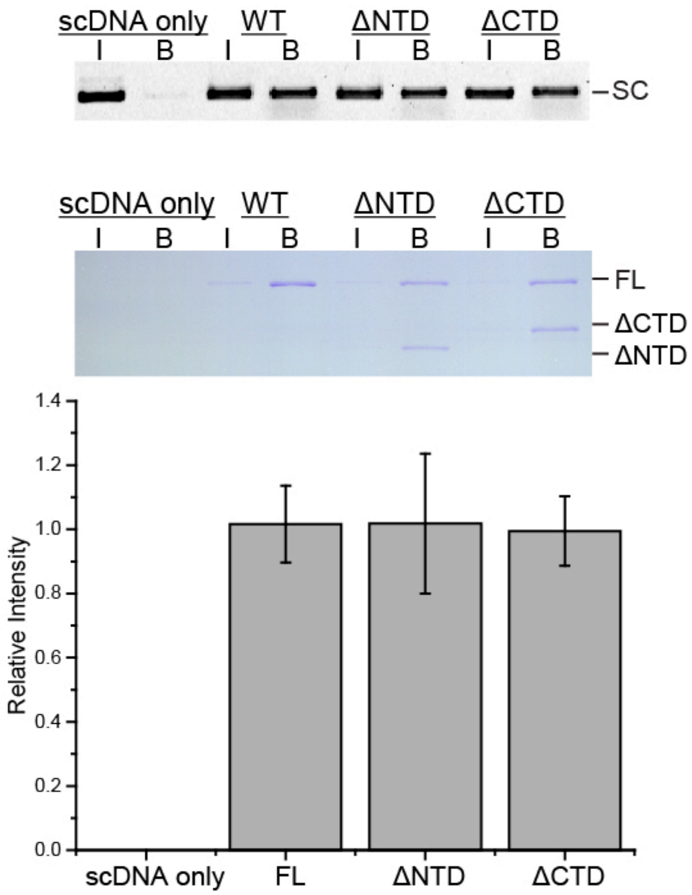

Supplementary Figure 3. Interaction of PFV intasomes with supercoiled plasmid DNA
PFV intasomes, including FL, ΔNTD, and ΔCTD, were assembled with biotinylated viral oligomers. The intasomes were incubated with supercoiled plasmid DNA and streptavidin conjugated magnetic beads. The plasmid DNA associated with the beads was analyzed by agarose gel electrophoresis with ethidium bromide staining (top panel). IN protein associated with the beads was analyzed by PAGE stained with Coomassie blue (center panel). The supercoiled plasmid precipitated by intasomes and beads was quantified and is expressed relative to PFV FL intasome (bottom graph). Experiments were performed at least two times with at least two independent intasome preparations. Error bars indicate standard deviation. I, 5% of the input supercoiled plasmid or intasomes. B, DNA or protein associated with streptavidin conjugated beads. SC, supercoiled plasmid.

Full Gel Images for Figure 2

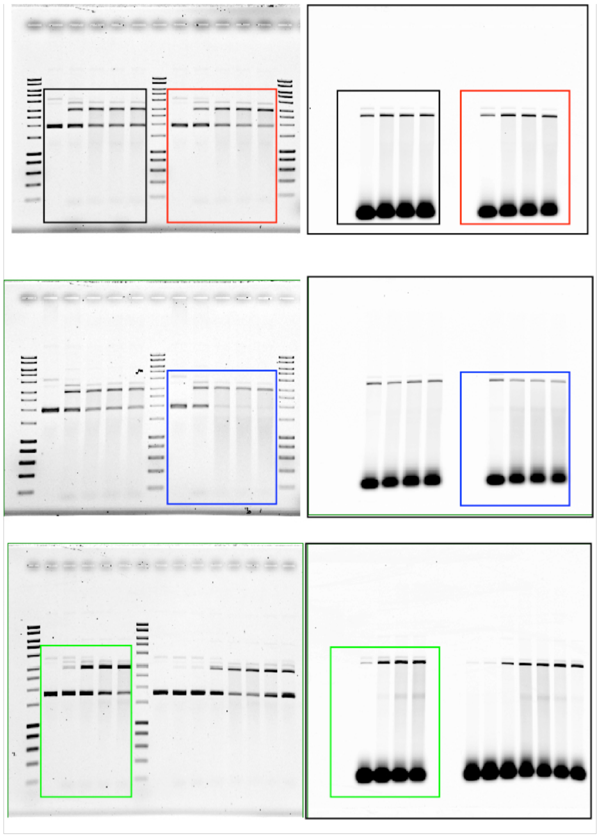

Ethidium stained gels are on the left column while Cy5 scanned gels are on the right. Black boxes denote FL lanes used. Red boxes denote ΔNTD lanes used. Blue boxes denote ΔCTD lanes used. Green boxes denote 300 mM NaCl lanes used.

Full Gel Images for Figure 3

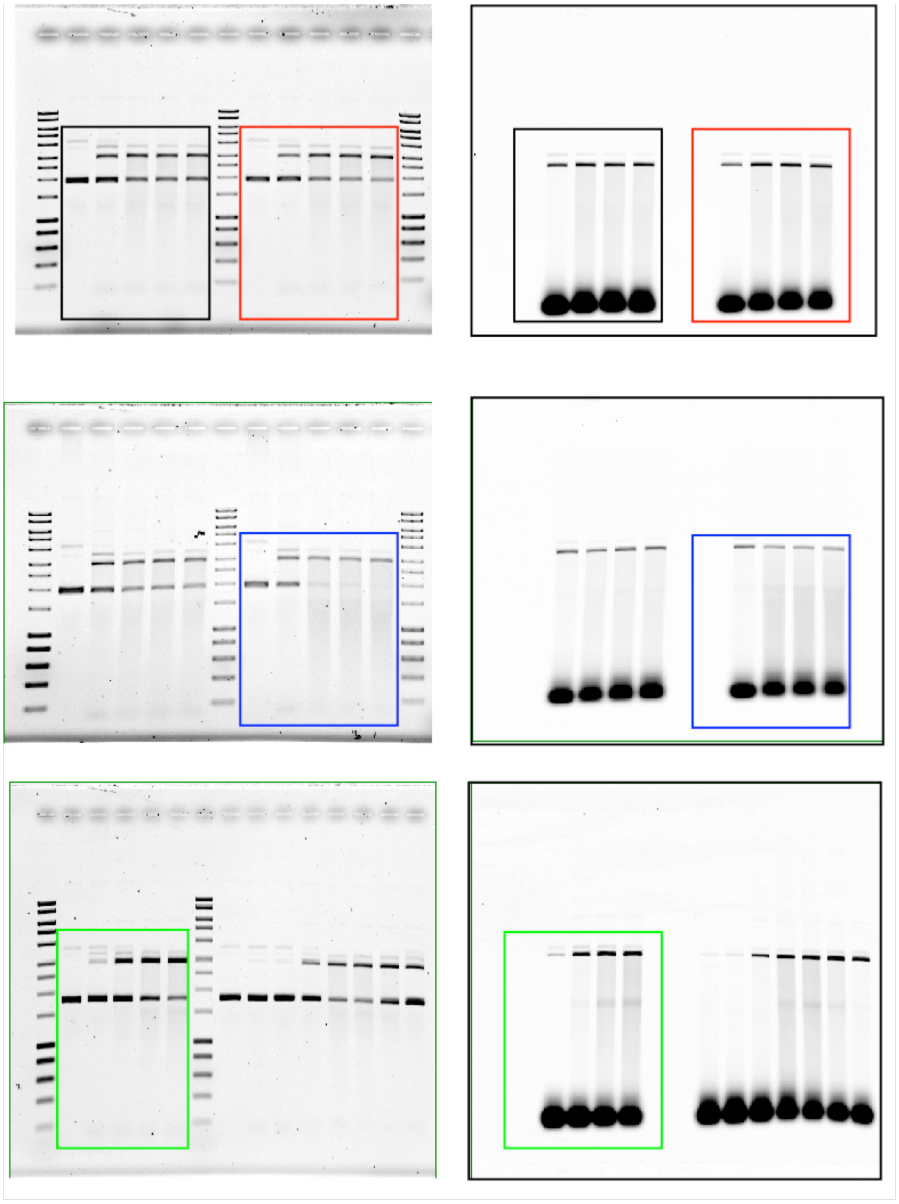

Ethidium stained gels are on the left column while Cy5 scanned gels are on the right. Black boxes denote FL lanes used. Red boxes denote ΔNTD lanes used. Blue boxes denote ΔCTD lanes used.

Full Gel Images for Figure 4

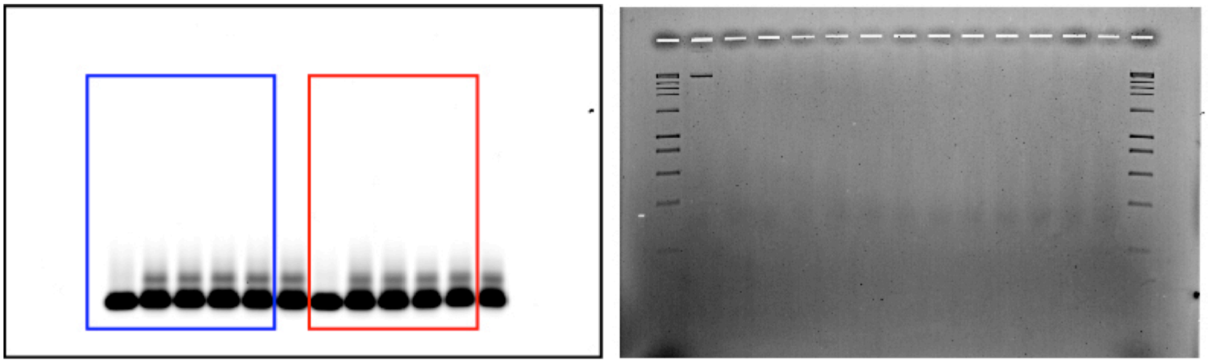

Blue and red boxes denote gels used in figure. Right panel is ethidium stain of same gel.

Full Gel Images for Figure 5

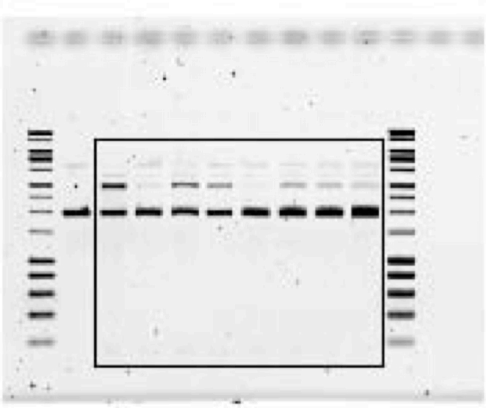

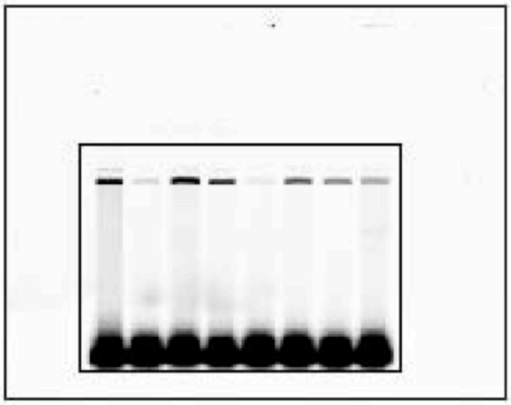

Ethidium stained gels are on the left column while Cy5 scanned gels are on the right. Black boxes denote lanes used.

Full Gel Images for Figure 6

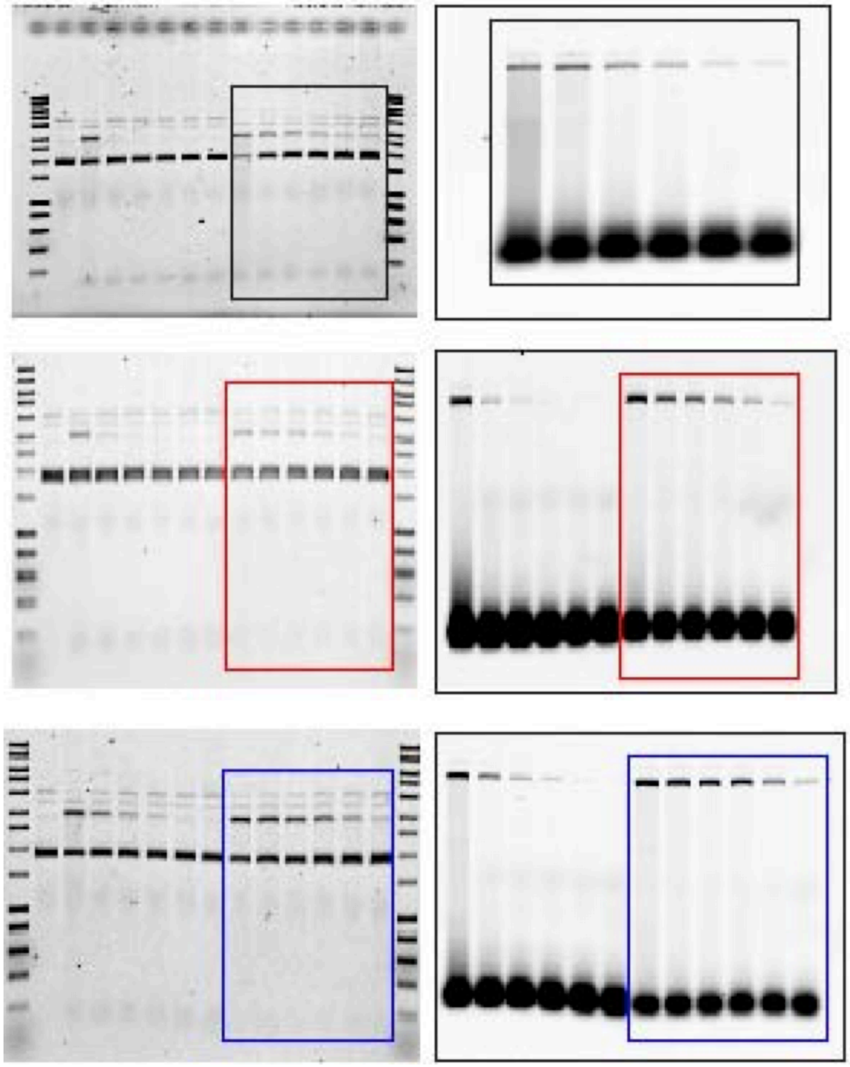

Ethidium stained gels are on the left column while Cy5 scanned gels are on the right. Black boxes denote FL lanes used. Red boxes denote ΔNTD lanes used. Blue boxes denote ΔCTD lanes used.

Full Gel Images for Figure 7

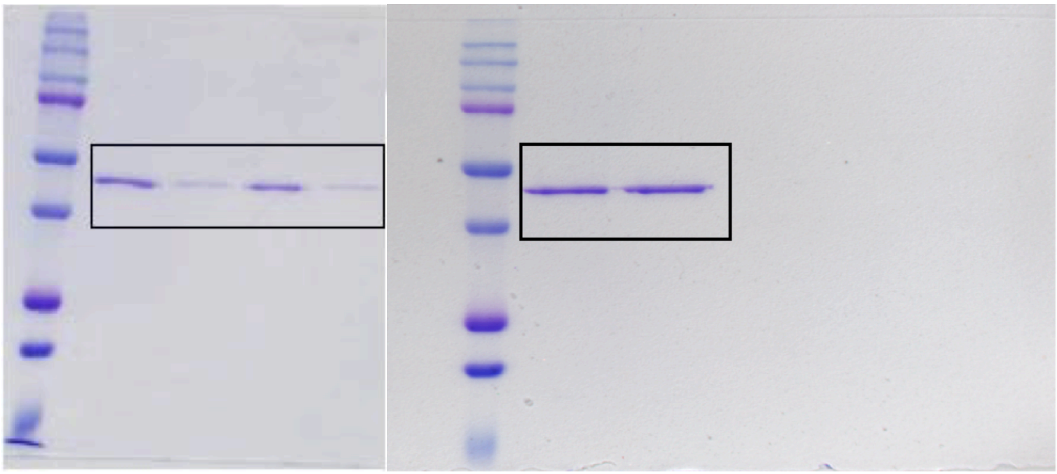

Black box denotes lanes used

Full Gel Images for Supplementary Figure 1

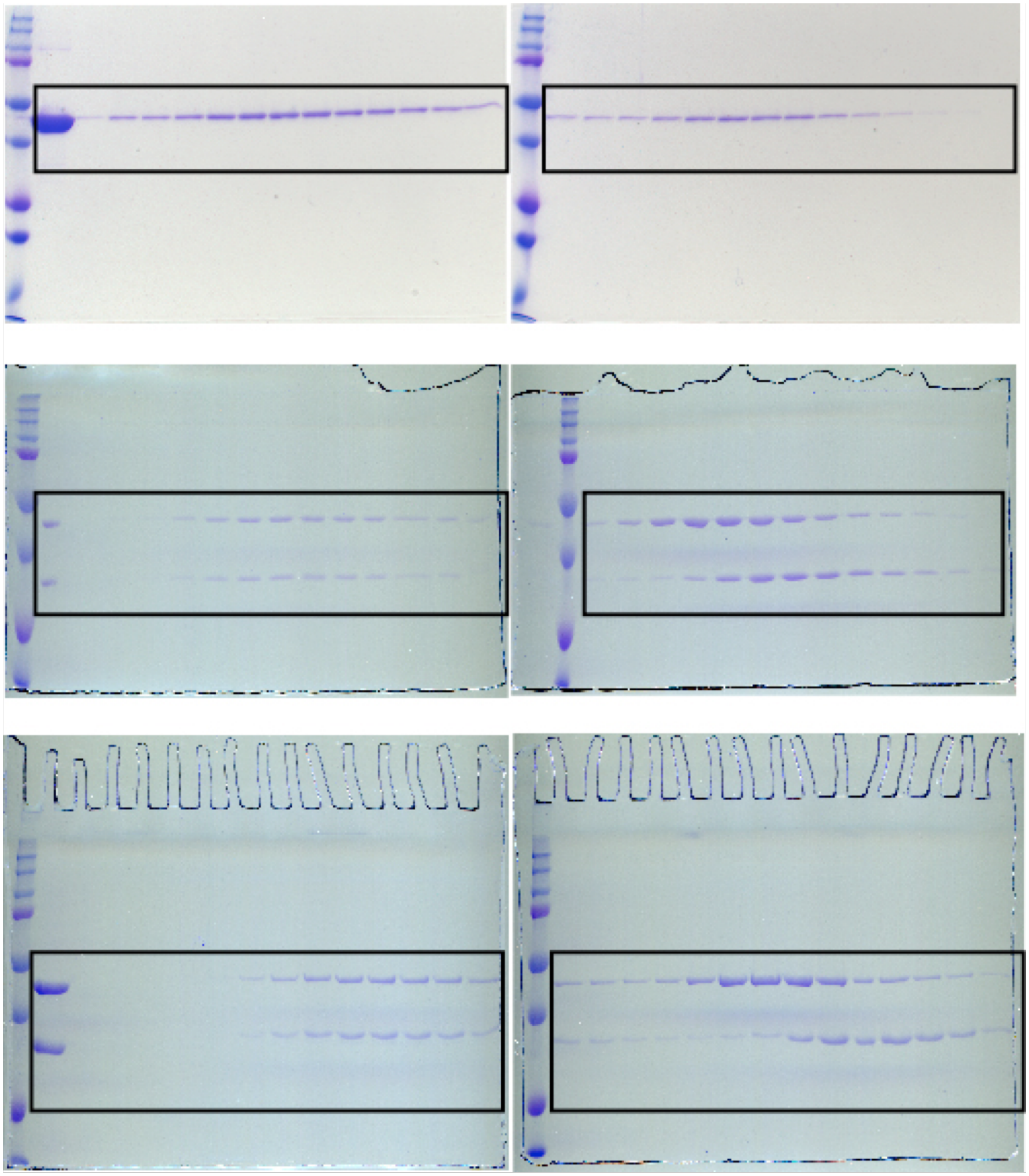

Black boxes denote lanes used.

Full Gel Images for Supplementary Figure 3

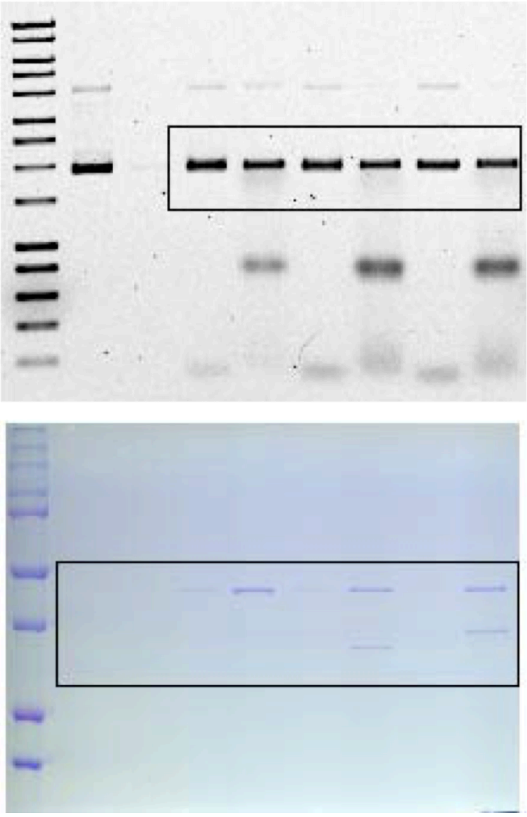

Ethidium stained gel is on the top panel while the acrylamide gel is on the bottom panel.

Black boxes denote lanes used.
